# Simulated microgravity and the antagonistic influence of strigolactone on plant nutrient uptake in low nutrient conditions

**DOI:** 10.1038/s41526-018-0054-z

**Published:** 2018-10-17

**Authors:** Guowei Liu, Daniel Bollier, Christian Gübeli, Noemi Peter, Peter Arnold, Marcel Egli, Lorenzo Borghi

**Affiliations:** 1 Institute of Plant and Microbial Biology, University of Zurich, Zollikerstrasse 107, 8008 Zurich, Switzerland; 2 Institute of Medical Engineering, HSLU Lucerne, Obermattweg 9, 6052 Hergiswil, Switzerland

## Abstract

Human-assisted space exploration will require efficient methods of food production. Large-scale farming in presence of an Earth-like atmosphere in space faces two main challenges: plant yield in microgravity and plant nutrition in extraterrestrial soils, which are likely low in nutrients compared to terrestrial farm lands. We propose a plant-fungal symbiosis (i.e. mycorrhiza) as an efficient tool to increase plant biomass production in extraterrestrial environments. We tested the mycorrhization of Solanaceae on the model plant *Petunia hybrida* using the arbuscular mycorrhizal fungus *Rhizophagus irregularis* under simulated microgravity (*s0-g*) conditions obtained through a 3-D random positioning machine. Our results show that *s0-g* negatively affects mycorrhization and plant phosphate uptake by inhibiting hyphal elongation and secondary branching. However, in low nutrient conditions, the mycorrhiza can still support plant biomass production in *s0-g* when colonized plants have increased SL root exudation. Alternatively, *s0-g* in high nutrient conditions boosts tissue-specific cell division and cell expansion and overall plant size in *Petunia*, which has been reported for other plants species. Finally, we show that the SL mimic molecule *rac-GR24* can still induce hyphal branching in vitro under simulated microgravity. Based on these results, we propose that in nutrient limited conditions strigolactone root exudation can challenge the negative microgravity effects on mycorrhization and therefore might play an important role in increasing the efficiency of future space farming.

## Plant biology: Plant hormone promotes beneficial interactions with fungi in microgravity

A plant hormone that regulates shoot branching and promotes symbiotic interactions with soil fungi can help crops overcome the negative effects of microgravity. A team led by Lorenzo Borghi from the University of Zurich, Switzerland, grew petunia flowering plants together with a beneficial fungus in simulated microgravity conditions. They showed that the lack of Earth-like gravity led to reduced production of mycorrhiza, a plant-fungal interaction known to help the plant absorb nutrients when low in soil. Plants that secrete high levels of this hormone, the carotenoid-derived strigolactone, can still thrive under microgravity conditions, as can fungi treated with a synthetic molecule that mimics the function of strigolactone. The findings could help inform best practices for space farming under the kinds of soil and environmental conditions present on space missions or on other planets.

## Introduction

Mycorrhizas are plant-fungal symbioses that occur in 95% of land plant families,^1^ among them the majority of staple crops. Through their fungal hyphae, mycorrhizas allow plants to enlarge their root system and reach eventually sparcely available nutrients such as phosphate, nitrogen, and micronutrients, as well as water. In turn, plants provide mycorrhizal fungi with sugars and lipids.^2^ This symbiosis is assumed to be advantageous for plant development, seed yield, and biomass accumulation when nutrient conditions are below optimal.^3^ The initiation of this symbiosis occurs with the plant root exudation of phytohormones belonging to the recently characterized strigolactone (SL) family.^4^ SLs are carotenoid derivatives that play several roles in regulating root and shoot architecture, biotic and abiotic stress resistance, and stimulate fungal hyphal branching toward the host plant root.^5^ As SL biosynthesis and transport are induced by low nutrient conditions, especially phosphate and nitrogen, SL signaling shapes plant development accordingly to the environmental conditions. SL biosynthesis and signaling seem to follow a rather linear pathway shared among many plant species.^6^ All-trans-β-carotenoids are converted to the bioactive SL precursor carlactone first by the enzymatic activities of the iron-containing protein DWARF27 (D27) and then by two carotenoid cleavage dioxygenases CCD7 and CCD8, respectively, decreased apical dominance3/DAD3 and decreased apical dominance1/DAD1 in *Petunia hybrida*.^7^ From carlactone, a plant-species-specific pool of P450 monooxygenases synthetizes SL molecules with different stereochemistry and decorations (e.g., strigol and orobanchol), which are most abundant in rice and *Petunia*^8,9^ or metyl-carlactonoic acid, isolated in *Arabidopsis*.^10^ SL signaling is detected by a heterodimeric receptor consisting of the alpha/beta hydrolase DWARF14 (D14) and the F-BOX protein—more axillary branches2 (MAX2). In the presence of SL, the two proteins assemble hydrolyzing SL. This signal starts a cascade of events leading to the ubiquitination of transcriptional repressors and to the activation of gene expression, e.g., ideal plant architecture1 (*IPA1*).^11^

SLs have several functions in planta, such as the regulation of lateral branching above ground and the already mentioned induction of fungal hyphal branching. SL transport and exudation are catalyzed through the ABCG class protein—pleiotropic drug resistance1^9^ from *P. hybrida*. Up-to-date, SL cellular exporters have been isolated only in Solanaceae^12^ but characterized only in *Petunia*. However, phylogenetic analyses^13^ reveal the presence of *PDR1* homologs in crops and Leguminosae. *P. hybrida* was chosen for this investigation on mycorrhization in simulated microgravity (*s0-g*) for its proximity to staple food plants like tomatoes, potatoes, and eggplants, and for the availability of mutant plants with altered SL transport efficiency. The latter include *pdr1 ko*^9^ and PDR1 over-expressor (PDR1 OE) plants,^14^ respectively, shown to be low or high in SL root exudation and root mycorrhization. Below ground, *PDR1* is expressed in root tips, where *DAD1* is also present, and in specialized, non-suberized root cortex cells named hypodermal passage cells (HPCs). HPCs are not only the exudation point for SL but also constitute the entrance gate for fungal hyphae into the plant root.^15^ A suberized hypodermis and HPCs are present in the majority of crops,^16^ therefore PDR1 and its homologs are assumed to have a key role in the regulation of plant nutrition even outside of the Solanaceae family.

To date, mycorrhization has not been assayed in space-like conditions, likely because environmental requirements are difficult to re-create on the International Space Station (ISS) and microgravity conditions are short-lived on parabolic flights. Recently, Dauzart et al.^17^ showed that nodulation of the legume model plant *Medicago truncatula* was affected on a two-dimensional (2D) clinostat, an alternative method of generating a simulated microgravity environment. They found that mycorrhization contributes to regulate nodulation in such conditions, but no mycorrhization data were provided. The efficiency of mycorrhization, and therefore of plant nutrient uptake, are important parameters to investigate before farming remote sites on the Moon or in space stations, where native or available soils might be extremely different from Earth^18^ and gravity forces are lower than on Earth or in the range of microgravity. The shipment of soil and fertilizer from Earth to space might be an expensive challenge in the long run. Instead, using lunar soil that represents a fine fraction of the native regolith might serve as basal growth medium for plants with the added help of mycorrhizal fungi. Studies have already been conducted in 2008 at the European Space Agency (ESA) where marigolds (*Calendula*) were cultivated on crushed rock that closely resembles lunar soil. In such or similar substrates, mycorrhization of plant roots could be vital for successful growth. Mycorrhization would also assure sustainable agriculture in space, a place where a balanced resource allocation and management are more essential than on Earth. Therefore, for the future of space-crop production, it is important to assess if mycorrhization takes place in microgravity or in altered gravity environments of long-term space stations, other planets or moons.

We hypothesized that microgravity, among the other extraterrestrial factors, might affect mycorrhization efficiency because SL biosynthesis and transport are positively regulated by the auxin signaling pathway.^19,20^ Auxin is a tryptophan-derived phytohormone that shapes several aspects of plant development. Its allocation in plant tissues is regulated by gravity, thus making auxin the main player in gravity sensing in plants, as reported in several scientific space missions.^21^ Currently, we know that microgravity influences auxin distribution and auxin transport in several plant species.^22^ We aim to test mycorrhization in wild-type plants and mutants for SL transport or synthesis under simulated microgravity conditions, and thus investigate not only the physical influence of microgravity on this plant-fungal symbiosis, but also to assay the efficiency of SL as a tool to promote mycorrhization under microgravity conditions. These results will establish and enhance our knowledge of mycorrhization in space and show that mycorrhiza could be a feasible and smart tool to increase plant adaptability and yield also in extraterrestrial environments.

## Results

**Fig. 1:**
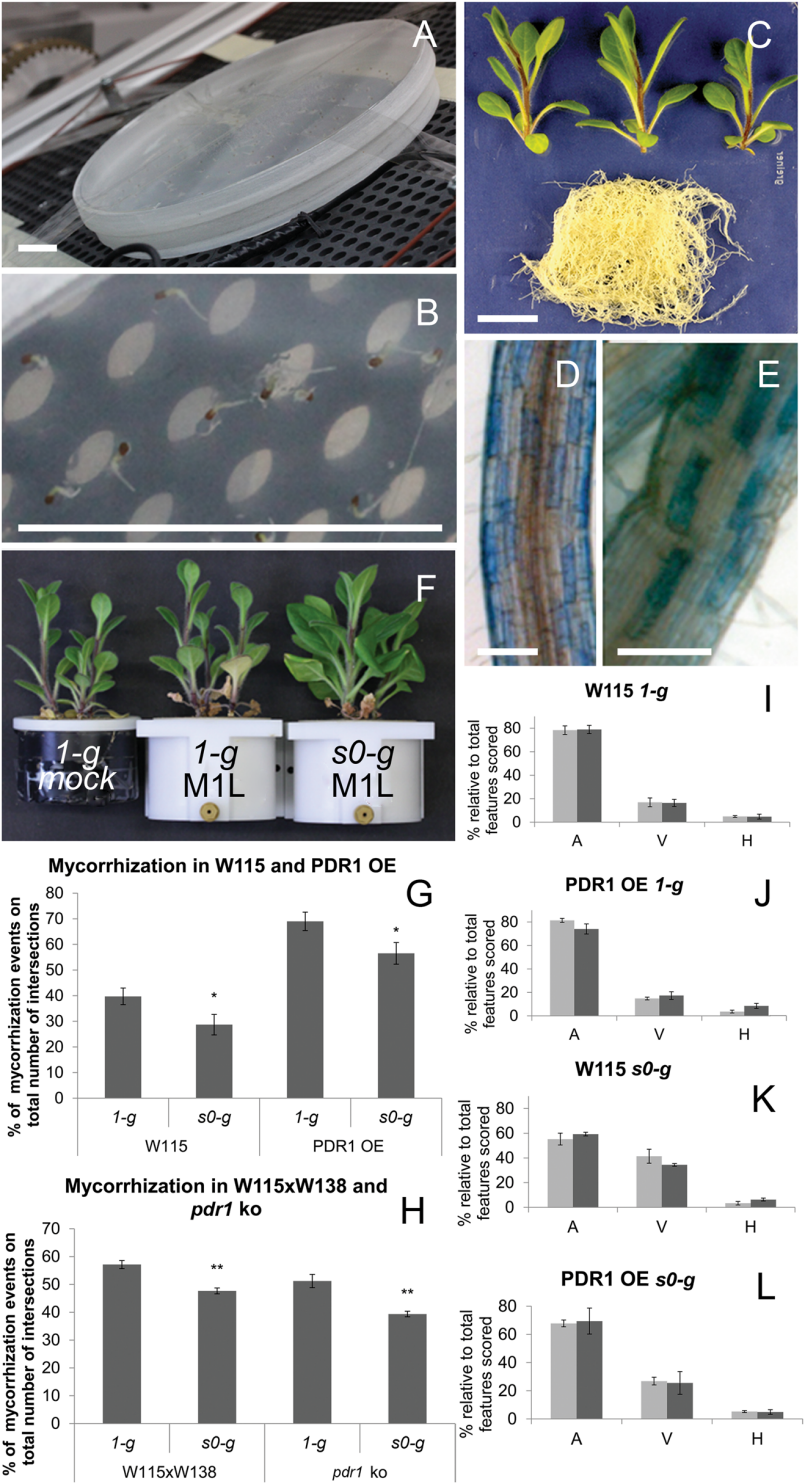
*Petunia* mycorrhization in simulated microgravity. **a** A Petri dish mounted on the RPM to test *Petunia* germination in *s0-g*. **b** *Petunia* seedlings germinated in *s0-g* show agravitropic radicle development. **c** A 40-day-old *pPDR1:GUS* plantlets (3×) grown in a M1L chamber: shoots (separated) and roots (pooled). **d**, **e** *pPDR1:GUS* expression pattern in main **d** and lateral **e** root grown in *s0-g*. **f** Comparison of plant growth at *1-g* in mock pots and at *1-g* and *s0-g* in M1L chambers. **g** Mycorrhization rates in WT and PDR1 OE plants grown at *1-g* and *s0-g*. **h** Mycorrhization rates in WT and *pdr1 ko* plants grown at *1-g* and *s0-g*. **i**–**l** Quantification of mycorrhizal structures relative to the total features scored. Black and gray bars represent 2 different replicates with each 300 root-grid intersections analyzed. Scale bars: **a** = 0.5 cm; **b**, **c** = 1 cm; **d**, **e** = 280 μm. Error bars are s.e.m. A arbuscules, V vesicles, H hyphae

The workshop of the Institute of Plant and Microbial Biology of the University of Zurich developed eight multigen-1-like (M1L) growth chambers to simulate plant growth conditions present on the ISS. These M1L chambers pave the way for future experiments on microgravity here on Earth. They were designed for easy mounting on a random positioning machine (RPM) (Supplementary Fig. 1)—a device that provides a simulated microgravity *0*-*g (s0-g*) environment to samples on ground.^23^ Because the results obtained by this device closely reflect the results from real microgravity exposure, the RPM has evolved to the method of choice for simulating microgravity (see more in Materials and methods, Microgravity simulating devices). *Petunia* germination and growth was carried on into a phytotron adapted to fit the RPM. In the same phytotron, not mounted on the RPM, *1-g* mock samples were grown to compare with *s0-g* grown plantlets (Supplementary Video 1). *Petunia* seedlings were initially germinated in *s0-g* in Petri dishes containing plant agar medium to test the efficiency of the RPM in this closed environment (Fig. 1a). Root growth was compared to *1-g* seedlings placed vertically in the growth chamber. The seedling radicles that emerged after germination in *s0-g* were agravitropic (Fig. 1b). After 14 days, the RPM conditions strongly affected *Petunia* root growth, which far from being straight as in *1-g* (Supplementary Fig. 2A) followed multiple-directional gravity vectors (Supplementary Fig. 2B) and confirmed the efficiency of this system to simulate *0-g* and to allow *Petunia* growth.

*Petunia* seeds were then germinated on agar medium at *1-g*, selected for equal developmental stage by morphology, as *Petunia* germination cannot be synchronized by stratification, and 1 week later transferred into M1L chambers, either mounted on the RPM or in *1-g*. Root and shoot phenotypes, biomass, phosphate, gene expression levels, and mycorrhization rates were quantified and compared for s*0-g* and *1-g*. Microgravity effects were tested in wild-type *Petunia* accessions W115 and W115xW138. This was necessary because *Petunia* mutants like *pdr1 ko* are obtained via the transposon-bearing ecotype W138.^24^ So far, this ecotype could not be transformed by the *Petunia* community (personal communication) and transgenes like PDR1 OE can be inserted only in accessions prone to transgenic insertion like W115. PDR1 OE plants, over-expressing PDR1 and reported to score higher mycorrhization than the wild-type^25^ and *pdr1 ko* mutants,^9^ were included in the experimental setup in order to investigate the role of SL exudation in *s0-g* conditions. First, we assessed growth adaptation of *Petunia* to M1L chambers since M1 chambers have only been used for ISS experiments on *Arabidopsis thaliana*,^26^ a well-known model plant that is not a host to mycorrhizal fungi. Continuous light conditions were chosen to accelerate plant growth and to allow the scoring of mycorrhization occurring 30 days after incubation (30 d.a.i.) with the fungus, compared to 60 d.a.i. under long-day conditions. The 30-day difference allowed us to increase the number of experiments and plants, the latter limited by the capacity of the RPM (8 M1L chambers per replica) and M1L chambers (3 *Petunia* plants per chamber). During the 30 days, M1L chambers were supplied with water alternated with a mycorrhizal inoculum buffer (Materials and methods). This is a good strategy to control the Pi amount in the soil and still supplying the required micro and macro nutrients to plants growing on clay-based substrates.^25^ Stem and root development of *Petunia* adapted to the small pot volumes (Fig. 1c) and dwarf phenotype did not affect the typical mosaic expression pattern of PDR1 in either the main or lateral roots,^9^ as visualized via the *pPDR1:GUS* reporter (Fig. 1d, e). Plant growth in M1L chambers or in mock pots (i.e., pots with the same soil and nutrient volume of M1L chambers but not built to be mounted on the RPM) showed no differences at *1-g* (Fig. 1f), thus allowing us to maximize plant growth in *s0-g*. A clay/soil mix solution, which is higher in nutrients than clay alone, (Liu et. al.^25^) was initially used for plant growth tests (Fig. 1f). It was replaced with clay, as in *s0-g* conditions, the soil mix liquefied after the first watering. Because it leaked out of the M1L chambers, it was not possible to quantify the amount of fungal spores present in the pot after each watering.

Mycorrhization levels of W115 (WT), PDR1 OE, W115xW138 (WT), and *pdr1 ko* plants were quantified (Materials and methods) for *1-g* and *s0-g* grown on clay + AMF inoculum. Equal inoculum amounts of the mycorrhizal fungus *Rhizophagus irregularis* were mixed with clay and aliquoted in M1L chambers, where *Petunia* seedlings were then transferred from plates. At *1-g*, PDR1 OE roots reached mycorrhization levels up to 69% of the root segments analyzed, confirming the previously reported high mycorrhization capacity^25^ despite these unusual growth conditions (Fig. 1g). In contrast, mycorrhization in W115 wild-type roots did not reach 40% (Fig. 1g). This trend occurred at *s0-g*, although both W115 and PDR1 OE plants were significantly less mycorrhized than at *1-g*, (see Materials and methods for statistical methods and Supplementary Table 1) reaching a maximum of 28.8% and 56.5% of root colonization, respectively (Fig. 1g). The same experimental setup with *pdr1 ko* and its wild-type background (W115xW138) plants showed that microgravity significantly decreased mycorrhization levels in both lines (Fig. 1h), especially in mutants for SL exudation, as expected. Surprisingly, mycorrhizal features, i.e., the relative amounts of arbuscule (the plant-fungal interface for the symbiotic nutrient exchange), vesicles, and hyphae changed between *1-g* and *s0-g* conditions. At *1-g*, 80% of the mycorrhiza consisted of arbuscules independent of the genotype (Fig. 1i, j and Supplementary Fig. 2C, D, G, I). At *s0-g*, PDR1 OE plants still harbored 75% of arbuscules, while in W115 arbuscules dropped below 60% and vesicles increased from 20 to 40% (Fig. 1k, l and Supplementary Fig. 2E, F, H, J).

**Fig. 2:**
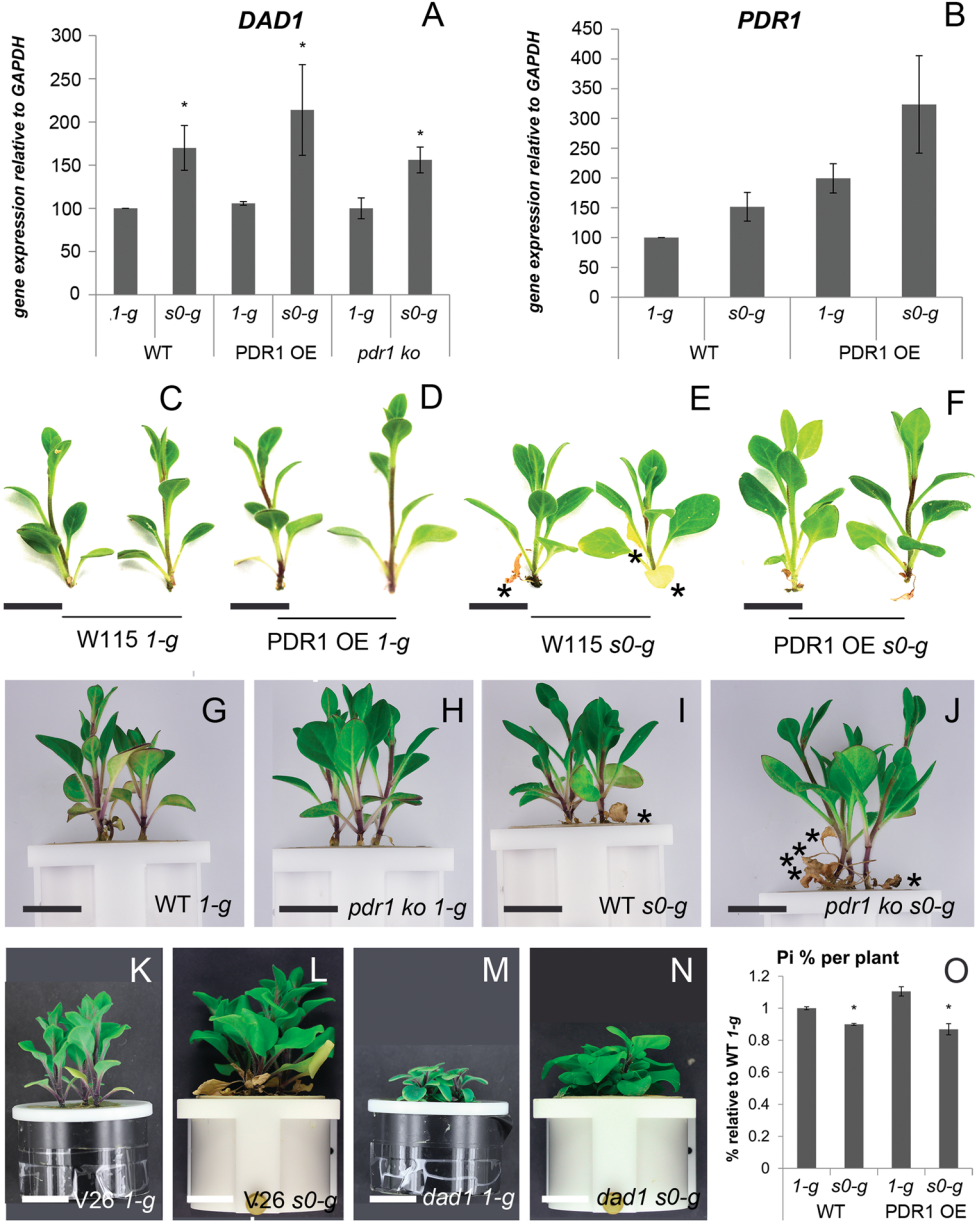
*Petunia* biomass production in simulated microgravity. **a**, **b** Gene expression quantification via qPCR of *DAD1* **a** and *PDR1* **b**. **c**–**f** Representative shoots of WT (W115) **c**, **e** and PDR1 OE **d**, **f** plants grown at *1-g* **c**, **d** and *s0-g* **e**, **f**. **g**–**j** Representative shoots of WT (W115xW138) **g**, **i** and *pdr1 ko* **h**, **j** plants grown at *1-g* **g**, **h** and *s0-g* **i**, **j**. **k**, **n** Representative shoots of V26 WT **k**, **l** and *dad1* plants **m**, **n** grown at *1-g* and *s0-g*. **o** Phosphate quantification relative to WT *1-g* conditions. Scale bars: **c**–**f** = 1.8 cm; **g**–**n** = 2.5 cm. Error bars are s.e.m.

The low-mycorrhization levels we observed in *s0-g* conditions were independent of the plant genotype and might be caused by decreased SL biosynthesis and/or exudation. Gene expression of the SL biosynthetic enzyme *DAD1* and of the SL transporter *PDR1* were determined by quantitative PCR*. DAD1* expression was induced by *s0-g* up to two folds compared to *1-g* samples (Fig. 2a). *PDR1* levels were slightly induced by *s0-g* (Fig. 2b), however not significantly. Interestingly, in *s0-g* conditions, *DAD1* expression levels of *pdr1 ko* plants were higher compared to *1-g* (Fig. 2a), suggesting that *DAD1* induction is caused by effects that are additive to the mutation in *pdr1*, which was previously reported to increase *DAD1* expression per se.^14^ *DAD1* and *PDR1* are both induced by auxin and microgravity is known to alter auxin distribution in plant roots.^22^ We propose that this effect might be present in *Petunia* and is responsible for the higher expression levels of the SL-related genes we analyzed.

**Fig. 3:**
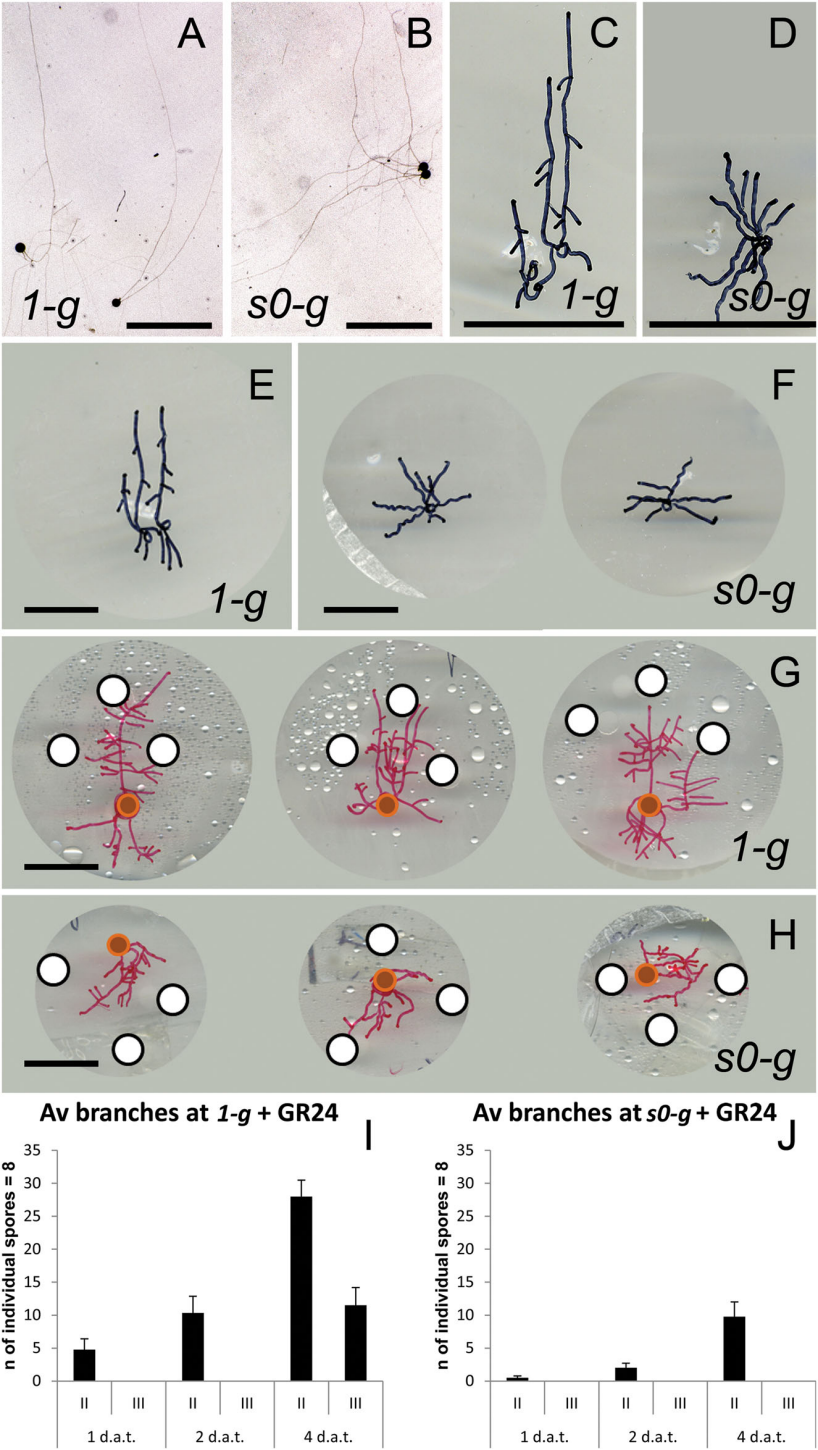
Hyphal elongation and branching in simulated microgravity. **a**–**d** Representative *R. irregularis* spores and hyphal branching at *1-g* and *s0-g*. **e**, **f** Mocks (minus *rac-GR24*) at *1-g* and *0-g* 4 days after germination. **g**, **h** *rac-GR24* added to filter paper pads. **i**, **j** Average amounts of branches for single spore at *1-g* and *s0-g*. Scale bars: **a**–**h** = 5 mm. Error bars are s.e.m.

SL biosynthesis and root exudation can be also positively regulated by environmental stimuli, such as low nutrient conditions, especially phosphate and nitrogen scarcity. The so-induced SL synthesis and exudation to the rhizosphere boost mycorrhization and extend plant nutrient scavenging to larger soil volumes.^27^ Plants grown in *s0-g* might experience starvation compared to *1-g* plants. Despite their higher expression of *DAD1* and *PDR1*, they obtain lower mycorrhization rates than plants at *1-g*. Also, wilting/dead leaves in each experimental setup were most abundant in *s0-g* low-mycorrhization conditions (Fig. 2e, i, j). Phosphate (Pi) quantification per plant confirmed that W115 and PDR1 OE plants grown in *s0-g* sequestrated less phosphate from soil than plants grown at *1-g* (Fig. 2o). Further, low mycorrhization is supported by the low Pi uptake. A possible explanation for the discrepancy between high SL signaling and low mycorrhization could be that the *s0-g* environment decreases the efficiency of the mycorrhiza, independent of the amount of SL exuded into the rhizosphere. Mycorrhizal hyphae can sense gravity and elongate in an anti-gravitropic way through a gravity sensing mechanism previously described.^28^ We then germinated *Rhizophagus irregularis* spores on a 2D clinostat placed into a fungal spore incubator (Materials and methods), to assay if gravity can affect hyphal elongation and therefore mycorrhization efficiency. Four days after germination, hyphal growth in clinostat-grown spores and *1-g* mock were compared, where the latter were grown vertically in the incubator. At *1-g*, fungal spores elongated single- or double-main hyphae that additionally generated secondary hyphae (Fig. 3a, c). On the clinostat, hyphal elongation was reduced and multi-directional with a lower amount of secondary hyphae detected (Fig. 3b, d): 10 ± 0.42 secondary hyphae per spore at *1-g* and 5.75 ± 1.18 at *s0-g*, respectively. The plate surface area, covered by the AMF body grown at *1-g*, was in average 1.7 ± 0.3 folds larger than in clinostat conditions. We applied *rac-GR24*, a SL mimic molecule, in *1-g* and *s0-g* to assay if the previously reported SL-induced hyphal branching^4^ was affected by changes in gravitational force. Four days after germination (Fig. 3e, f), the fungal spores were temporarily removed from the clinostat or from the fungal incubator to apply *rac-GR24* (Materials and methods). At *1-g*, *rac-GR24* induced an average of 10.3 ± 2.5 new hyphal branches 2 days after treatment (d.a.t.) (Fig. 3g, i) and only 2 ± 0.7 at *s0-g* (Fig. 3h, j). Four d.a.t., fungi at *1-g* developed tertiary and higher orders of branching (Fig. 3i), but not at *s0-g* (Fig. 3j). These results show that main hyphal elongation and secondary hyphal branching of *R. irregularis* are negatively regulated by simulated microgravity and that microgravity could therefore affect the development of the mycorrhiza by reducing the possibility of physical interaction between fungal hyphae and plant roots.

**Fig. 4:**
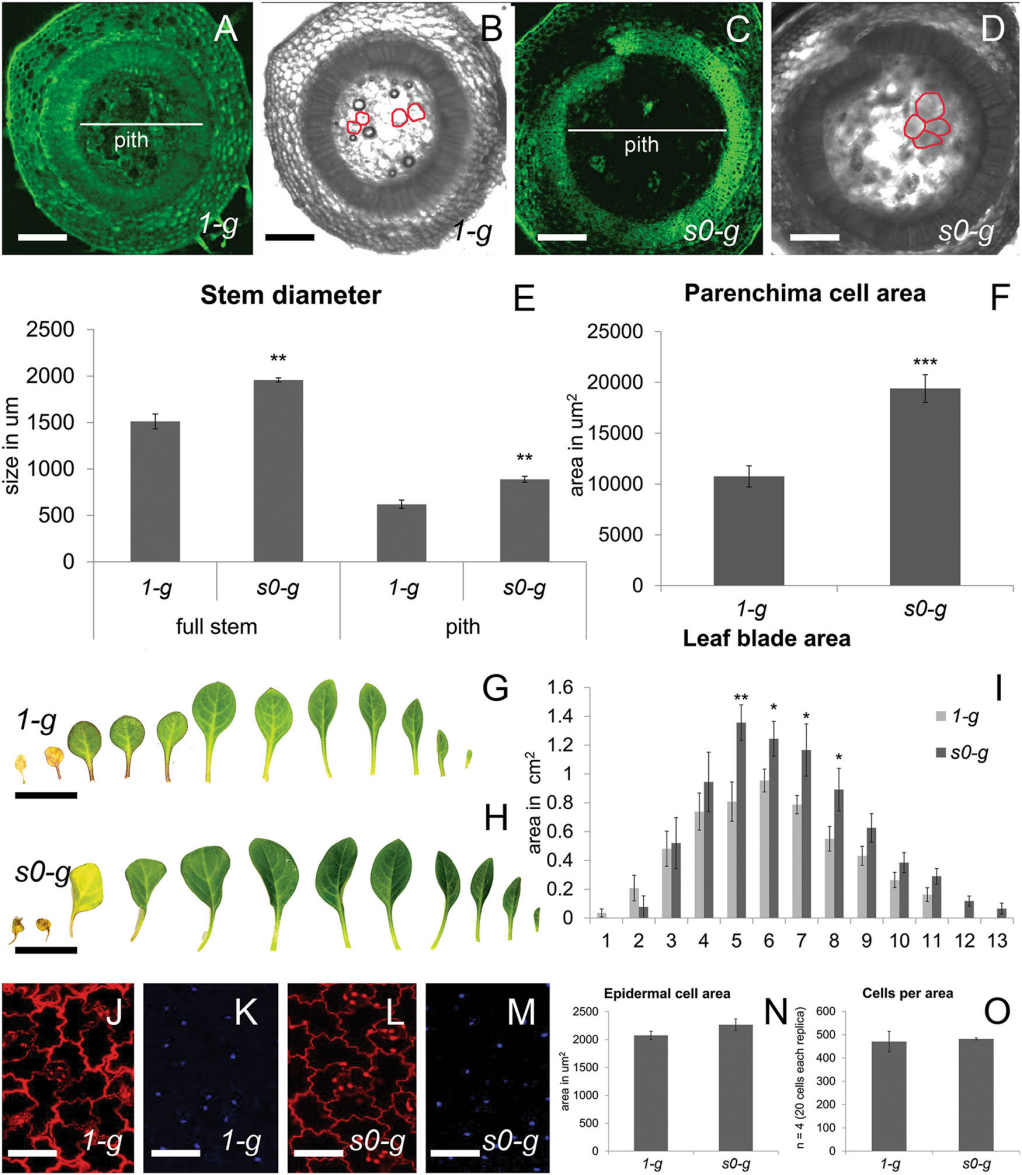
*Petunia* development in simulated microgravity. **a**–**d** Autofluorescence and transmitted light detected via confocal laser scanning microscopy. Stem sections of wild-type plants at node 4: **a**, **b** *1-g*; **c**, **d** *s0-g*. In red, the boundary of representative pith cells. **e** Quantification of full stem and pith diameter. **f** Area of parenchima cells from the pith. **g**, **h** Leaves of wild-type W115 plants grown, respectively, at *1-g* and *s0-g*. **i** Quantification of leaf blade area in wild-type plants at *1-g* and *s0-g*. **j**–**m** Propidium iodide staining of leaf 5 adaxial side (**j**, **l**) and DAPI staining (**k**, **m**) from plants grown at *1-g* (**j**, **k**) and *s0-g* (**l**, **m**). **n**, **o** Quantification of epidermal cell areas and cell per area in adaxial leaf sides. Scale bars: **a**–**d** = 260 μm; **g**, **h** = 1.4 cm; **j**–**m** = 45 μm. Error bars are s.e.m.

Plant size is clearly induced by *s0-g* conditions, compare, e.g., Fig. 2c, e; Fig. 2d, f; Fig. 2k, l; Fig. 2m, n. Microgravity has been previously reported to induce cell expansion in *Arabidopsis* hypocotyls through a mechanism that changes cell-wall plasticity^29^, and cell division.^30^ We hypothesized that similar mechanisms might be responsible for the thicker stems (Fig. 4a–e) and the larger leaves (Fig. 4g–i) we observed in *s0-g*. Cell size in transversal stem sections indicated that pith parenchyma cells were two folds larger in *s0-g* compared to *1-g* (Fig. 4f). Instead, leaf epidermal cells showed the same size and density in *1-g* and *s0-g* conditions (Fig. 4j–o), despite that leaf surface areas were clearly induced by *s0-g*. These results suggest that cell division also plays an important role in *s0-g*, at least for leaf biomass. Similarly below ground, root surfaces increased when grown in microgravity (Supplementary Fig. 2K–M). Large root volumes allow plants to scavenge nutrients more efficiently and might release plants from the option of mycorrhizal support, which is costly in terms of photosynthetic sugars.^31^ Still, despite their increased root volumes, *s0-g* plants are Pi starved, showing that in these growth conditions, mycorrhization is more efficient than an enlarged root system for scavenging Pi. Along with the increase in plant volumes was higher biomass accumulation in *s0-g* (Supplementary Fig. 2N). Interestingly, the largest increases in biomass production in *s0-g* were in PDR1 OE, V26, and *dad1* plants, which reached up to 60% higher biomass compared to the corresponding *1-g* mock plants. V26 and *dad1* plants (Fig. 2k–n) were grown on the above mentioned liquefied soil + clay mix. The higher nutrient conditions compared to clay + AMF inoculum^25^ might have allowed these plants to more efficiently use the expanded root system induced by *s0-g*. PDR1 OE plants had larger than wild-type roots (Supplementary Fig. 2M) and higher mycorrhization rates as shown above, thus giving them an advantage in nutrient uptake compared to wild-type. No significant changes were found between FW/DW ratios in W115 and PDR1 OE plants grown at *1-g* and *s0-g*: 89.95% ± 0.16 of shoot water content and 93.67% ± 0.29 of root water content independent of genotypes/gravity conditions. Therefore, we propose that the tissue-specific cell division and cell expansion were responsible for the differences in biomass production and were induced by microgravity.

## Discussion

We have shown how simulated microgravity force negatively influences the formation of mycorrhiza. We assume that a previous study on plant-microbe interactions in low gravity^17^ showed no differences in the mycorrhization rates of *Medicago truncatula* grown either at *1-g* or on a clinostat because growth conditions were set for nodulation, but were not suitable to investigate mycorrhization. Also, the quantification of mycorrhization in *M. truncatula* was recently proven to be difficult because of the high variability between mycorrhization rates in single plants.^32^ Mycorrhizal symbiosis is assumed to give a selective advantage by boosting plant nutrient uptake, especially in nutrient depleted conditions. Future space farmers could meet such conditions either for long-term space flights or for the colonization of new planets: that is precisely why mycorrhization efficiency was assayed in low nutrient and different gravity conditions. We have also shown that *Petunia* development is possible in our *s0-g* M1L chambers: in 30 days, plant could develop expanded leaves, elongated stems, and a root system. No lateral branches and no flowering transition were observed, possibly because of the limited nutrient and time duration. Still we could quantify a functional AMF colonization of *Petunia* roots, as fungal arbuscules were detected in root cortex cells. We showed that simulated microgravity conditions negatively affected the fungal hyphal development: hyphal elongation and branching were inhibited when fungal spores were germinated on a clinostat. The negative effects we observed on hyphal branching likely influenced the higher vesicle/arbuscule ratio in plants grown on our RPM, as arbuscule are structures made of repeated branching events, although several other plant factors are known to coordinate arbuscule formation.^33^ The high number of vesicles we quantified in mycorrhiza in *s0-g*, particularly in W115 WT background, suggests a low functional mycorrhization. This hypothesis is supported by the low Pi content for the same plants compared to *1-g* mocks.

Tips of fungal hyphae contain a gravity sensor made of oil droplets and protein crystals that was previously shown to sense even short-lived changes in microgravity, such as the ones obtained on parabolic flights.^34^ We propose that clinostat and RPM conditions do not allow hyphal gravitropic growth and slow down or inhibit the formation of higher branching orders, thus reducing mycorrhization efficiency. Lateral branching in fungal hyphae occurs only when a potential branching site is sufficiently far from the hyphal tip to break apical dominance through unknown mechanisms.^35^ A possible explanation for the rare hyphal branching in microgravity and for the low sensitivity to exogenous *rac-GR24* that we observed may result from the inhibition of hyphal extension: at *s0-g*, hyphal length is approximately half compared to *1-g* conditions. Interestingly, a localized GR24 source (Fig. 3g, h) could still stimulate some directional growth and hyphal branching despite *s0-g*. These results support the hypothesis that PDR1 OE plants in *s0-g* could still induce high mycorrhization levels because of enhanced SL exudation.^25^ PDR1 OE plants could not only be a useful tool to improve plant biomass production on Earth, but also in space and on other planets. Additionally, in space, the presence of detrimental parasitic weeds, whose germination is also induced by SL^36^ could be easily controlled or excluded. Finally, simulated microgravity did not affect the strong PDR1 OE mycorrhization performance compared to the wild-type, showing that SL exudation can be still modulated by increasing PDR1 expression despite the *s0-g* environment.

There was no significant difference in mycorrhization rates between wild-type and *pdr1* ko mutants grown either at *s0-g* or *1-g*, despite that the mutant plants do not exude SLs and were previously reported to mycorrhize very little, similar to *dad1* mutants.^9^ We suggest that the small pot volume of M1L chambers increased the chance of contact between fungal hyphae and plant roots even with low to no contribution from SL. Still, *s0-g* conditions can equally decrease mycorrhization efficiency in wild-type and *pdr1 ko* plants, showing that not only SL exudation into the rhizosphere, but also gravity sensing on the fungal side are important for the successful establishment of the mycorrhiza.

Despite the low mycorrhization and the decreased Pi uptake, plants grown in *s0-g* produced higher biomass than at *1-g*. A biomass increase up to 40% was reported in roots and hypocotyls of *Veronica arvensis* grown on a clinostat.^37^ Lateral root growth and hypocotyl elongation were found to be induced in clinostat conditions. The initiation of lateral roots is regulated by auxins^20^ and a re-distribution of the auxin transporter PIN1 due to microgravity was recently reported in cucumber roots.^22^ As auxin transport and synthesis are strongly conserved among land plant species,^20^ we propose that the observed plant architecture changes in *Petunia* grown in *s0-g* were also caused by auxin re-allocation.

The increase in plant biomass reported here in *s0-g* might be a result of an increased cell-cycle activity and consequently the generation of larger tissues (like in leaf epidermal cells) or of a stronger cell expansion (like in pith cells) regulated by hormonal signaling. The results of our experiments show that *s0-g* can induce tissue-specific cell division and cell expansion, possibly by cell-wall weakening as previously reported^38^ and through yet unknown, auxin-mediated effects on the cell cycle in shoot and root meristems^30^ that span from different distribution of cell-cycle phases to epigenetic modifications.^39^

To summarize, simulated microgravity reduced mycorrhization rates and Pi uptake in *Petunia*, likely because of inhibited hyphal growth. Additionally, the induction of root growth observed in *s0-g* plants might have also contributed to a decrease of the costly mycorrhization. On the other hand, microgravity could induce biomass production when plants were grown in nutrient-rich soil conditions or in limited nutrient soil if plants could perform high SL root exudation, like PDR1 OE plants. The accumulation of plant biomass in nutrient scarce soils driven by microgravity plus enhanced SL exudation will have to be assessed in staple food plants either on an Earth-bound RPM or on the International Space Station. Instead of transgenic PDR1 OE crops, natural accessions high in SL root exudation should be isolated and included in these investigations. Instead of clay, mimics for extraterrestrial soils should be generated not only to investigate their effect on plant nutrition, but also to test the adaptability of terrestrial AMF to novel alien conditions. Recent developments of mycorrhization research, focused on desert farming, showed how soil shapes mycorrhizal populations and the low adaptability of AMF strains to different niches.^40^ Still, the results presented here let us envisage a positive future for space-crop production, thanks to plant adaptability to microgravity regulated by phytohormonal signaling and plant-fungal symbiosis.

## Materials and methods

### Plant growth

*Petunia hybrida* plants var. Mitchell (W115) PDR1 OE,^14,25^ *pdr1 ko*, *dad1*, *pPDR1:GFP-PDR1*, *pPDR1:GUS*^9^ and relative wild-type backgrounds were germinated on plates at *1-g* and 5 days later selected for transferring in M1L chambers or mock pots. Growth was carried on at 24 h light conditions, 60% humidity, 25 °C for 30 days on clay (Oil dri, Chicago, USA) + *R. irregularis* inoculum kindly supplied by Professor Dr. Marcel van der Heijden (Agroscope, Zurich, Switzerland). M1L chambers and mock pots were supplied twice a week with ½ Murashige and Skoog Basal Salt Macronutrient (MS) solution with low phosphate as in Liu et al.^25^

### Microgravity simulating devices

#### Random positioning machine

Samples on the RPM turn constantly around two axes (60°/s). The motion pattern is programmed so that the gravity pull, which is working on the samples, is distributed spatially and temporally equally. The result is that the gravity vector mathematically averages to zero over time. In addition, the constant re-orientation of the gravity vector makes it impossible for biological systems to adjust to gravitational force, thus the resulting response is compatible to the response achieved by actual microgravity exposure. The results obtained on the RPM are generally in a good to fair agreement to actual space flown experiments of various cell types.^41^ Actually, the RPM has been established as method of choice for plant experiments under simulated microgravity conditions several years ago.^42^ In this study, the plastid position in columella cells of *Arabidopsis* seedlings had equal grown rates at low Earth orbit on board the Space Shuttle and on the RPM. This result, supported by the extensive work of Hoson et al.^43,44^ encourages the use of the RPM for plant experiments under simulated microgravity conditions. Today, plant scientists interested in gravitational aspects of plant physiology are still applying RPM conditions to simulate microgravity.^45^ The RPM, however, is just one method among others to create a microgravity-like environment and none of these replace microgravity achieved during space flights. It is also obvious that the RPM needs to be operated carefully, otherwise artifacts are introduced, which could lead to false-positive/-negative results. We have thus investigated the appropriate RPM settings in order to claim a microgravity-like environment^46^ and have formulated these specific recommendations.^41^ All our microgravity studies using the RPM follow these guidelines to ensure high-quality data.

#### Clinorotation

This instrument applies a similar principle as the RPM for generating a microgravity-like environment. The moving pattern however focuses on one axis only. The effects of gravity on the fungal spores are negated on clinostats by placing the samples in the center of the rotating horizontal axis for an extended period of time.

### GUS staining, PI staining, and DAPI staining

Beta-D-glucuronosideglucuronosohydrolase (GUS) staining of HPCs, propidium iodide (PI) staining of epidermal leaf cells and nucleic acid DAPI staining were performed as reported in Kretzschmar et al.^9^ and Sasse et al.^14^ with no changes. Briefly, fresh roots were incubated overnight in a GUS solution buffer and visualized with a light microscope without destaining. Fresh leaves were mounted for analysis on a Leica SP5 confocal laser scanning microscope and a drop of PI (1 μg/ml) positioned in the middle of the leaf blade. After 5 min, the signal was detected (excitation 488 nm/emission 617 nm). Same procedure was used for DAPI staining, but with different laser wavelengths (excitation 340 nm/emission 488 nm).

### Mycorrhization staining and scoring

Mycorrhization and mycorrhizal structures were ink stained and quantified as described in Akiyama et al.^4^ at a stereoscopic microscope. Briefly, roots were collected, fixed in potassium hydroxide 5% w/v, boiled for 10 min in water, and destained in acetic acid 5% v/v. We quantified the mycorrhizal structures with 500 root-grid intersections per sample per replicate. Mycorrhizal features (arbuscules, vesicles, and hyphae) were visually recognized with a stereoscopic microscope.

### Gene expression analysis

Real time PCR analysis was used to quantify the expression levels of *DAD1* and *PDR1*. Protocol and primer sequences are from refs.^14,25^ with no changes.

### Fluorescent and light microscopy

Confocal laser scanning microscopy and light microscopy as in Sasse et al.^14^ but with minor changes in tissue preparation for stem analyses. Approximately 1 cm long fresh stem segments were included in 4% agarose w/v. Hundred-micrometer-thick stem slices were immediately prepared with the aid of a vibratome for further analysis at the fluorescent and light microscope. A wavelength of 488 nm for excitation and an emission window peaking at 514 nm were set for fluorescence detection.

### Pi quantification

Phosphate extraction and quantification were performed as described in Liu et al.^25^ Nine plants were pooled per line per replica.

### Digital image quantification

Tissues for surface measurements were digitally acquired via scan or camera and areas were digitally quantified using ImageJ.

### Arbuscular mycorrhizal fungi growth on clinostat

*R. irregularis* spores were mildly surface sterilized (0.1% bleach v/v and 0.01% Triton-X v/v) for 3 min and rinsed with sterilized water 5 times. One or two spores were placed on Phytagel (Sigma-Aldrich, Buchs, Switzerland) plates (3.5 g/l), which were first sealed with Micropore paper tape (3 M, Rüschlikon, Switzerland) and then taped on a clinostat positioned at 32 °C, 4% CO_2_ v/v constantly rotating at 40 ° per s. *1-g* plates were vertically positioned on the floor of the incubator. Four days later, the germinated mycorrhizal fungi were observed at the microscope. Sterile filter paper pads soaked with 100 pg *rac-GR24* (Chiralix, Nijmegen, Netherlands) were added as shown in Akiyama et al.^4^

### Statistical analyses

Student’s *t*-test was applied to evaluate the significance of the presented results. *n* values and *p* values presented in Supplementary Table 1. Locations of M1L chambers and mock pots were randomized on the RPM and in the growth chamber, respectively.

## Electronic supplementary material

Supplementary Video 1

Supplemental Material

## Data Availability

The data that support the findings of this study are available from the corresponding author upon reasonable request.

## Electronic supplementary material

**Supplementary information** accompanies the paper on the *npj Microgravity* website (10.1038/s41526-018-0054-z).

## References

[1] Newman EI, Reddell P (1987). The distribution of mycorrhizas among families of vasculature plants. New Phytol..

[2] Rich MK, Nouri E, Courty PE, Reinhardt D (2017). Diet of arbuscular mycorrhizal fungi: bread and butter?. Trends Plant Sci..

[3] Gianinazzi S (2010). Agroecology: the key role of arbuscular mycorrhizas in ecosystem services. Mycorrhiza.

[4] Akiyama K, Matsuzaki K, Hayashi H (2005). Plant sesquiterpenes induce hyphal branching in arbuscular mycorrhizal fungi. Nature.

[5] Al-Babili S, Bouwmeester HJ (2015). Strigolactones, a novel carotenoid-derived plant hormone. Annu. Rev. Plant Biol..

[6] Lopez-Obando M, Ligerot Y, Bonhomme S, Boyer FD, Rameau C (2015). Strigolactone biosynthesis and signaling in plant development. Development.

[7] Napoli C (1996). Highly branched phenotype of the petunia dad1-1 mutant is reversed by grafting. Plant Physiol..

[8] Xie X (2013). Confirming stereochemical structures of strigolactones produced by rice and tobacco. Mol. Plant.

[9] Kretzschmar T (2012). A petunia ABC protein controls strigolactone-dependent symbiotic signalling and branching. Nature.

[10] Seto Y (2014). Carlactone is an endogenous biosynthetic precursor for strigolactones. Proc. Natl Acad. Sci. USA.

[11] Song X (2017). IPA1 functions as a downstream transcription factor repressed by D53 in strigolactone signaling in rice. Cell Res..

[12] Xie X (2015). Cloning and characterization of a novel *Nicotiana tabacum* ABC transporter involved in shoot branching. Physiol. Plant.

[13] Borghi L, Liu GW, Emonet A, Kretzschmar T, Martinoia E (2016). The importance of strigolactone transport regulation for symbiotic signaling and shoot branching. Planta.

[14] Sasse J (2015). Asymmetric localizations of the ABC transporter PaPDR1 trace paths of directional strigolactone transport. Curr. Biol..

[15] Sharda JN, Koide RT (2008). Can hypodermal passage cell diSstribution limit root penetration by mycorrhizal fungi?. New Phytol..

[16] Shishkoff N (1987). Distribution of the dimorphic hypodermis of roots in angiosperm families. Ann. Bot..

[17] Dauzart, A. J. C., Vandenbrink, J. P. & Kiss, J. Z. The effects of clinorotation on the host plant, *Medicago truncatula*, and its microbial symbionts. *Front. Astronom. Space Sci.* **3** (2016) https://www.frontiersin.org/articles/10.3389/fspas.2016.00003/full.

[18] Wamelink GW, Frissel JY, Krijnen WH, Verwoert MR, Goedhart PW (2014). Can plants grow on Mars and the moon: a growth experiment on Mars and moon soil simulants. PLoS ONE.

[19] Borghi L, Liu GW, Emonet A, Kretzschmar T, Martinoia E (2016). The importance of strigolactone transport regulation for symbiotic signaling and shoot branching. Planta.

[20] Vanneste S, Friml J (2009). Auxin: a trigger for change in plant development. Cell.

[21] Kittang AI (2014). Exploration of plant growth and development using the European Modular Cultivation System facility on the International Space Station. Plant Biol..

[22] Yamazaki C (2016). The gravity-induced re-localization of auxin efflux carrier CsPIN1 in cucumber seedlings: spaceflight experiments for immunohistochemical microscopy. NPJ Microgravity.

[23] Wuest SL (2014). A novel microgravity simulator applicable for three-dimensional cell culturing. Microgravity Sci. Technol..

[24] Koes R (1995). Targeted gene inactivation in petunia by PCR-based selection of transposon insertion mutants. Proc. Natl Acad. Sci. USA.

[25] Liu G (2018). Changes in the allocation of endogenous strigolactone improve plant biomass production on phosphate-poor soils. New Phytol..

[26] Link BM, Busse JS, Stankovic B (2014). Seed-to-seed-to-seed growth and development of *Arabidopsis* in microgravity. Astrobiology.

[27] Bouwmeester HJ, Roux C, Lopez-Raez JA, Becard G (2007). Rhizosphere communication of plants, parasitic plants and AM fungi. Trends Plant Sci..

[28] Grolig F, Doring M, Galland P (2006). Gravisusception by buoyancy: a mechanism ubiquitous among fungi?. Protoplasma.

[29] Soga K, Wakabayashi K, Kamisaka S, Hoson T (2002). Stimulation of elongation growth and xyloglucan breakdown in *Arabidopsis* hypocotyls under microgravity conditions in space. Planta.

[30] Medina FJ, Herranz R (2010). Microgravity environment uncouples cell growth and cell proliferation in root meristematic cells: the mediator role of auxin. Plant Signal. Behav..

[31] Walder F (2012). Mycorrhizal networks: common goods of plants shared under unequal terms of trade. Plant Physiol..

[32] Dreher D, Yadav H, Zander S, Hause B (2017). Is there genetic variation in mycorrhization of *Medicago truncatula*?. PeerJ.

[33] Luginbuehl LH, Oldroyd GED (2017). Understanding the arbuscule at the heart of endomycorrhizal symbioses in plants. Curr. Biol..

[34] Galland P (2014). The sporangiophore of *Phycomyces blakesleeanus*: a tool to investigate fungal gravireception and graviresponses. Plant Biol..

[35] Harris SD (2008). Branching of fungal hyphae: regulation, mechanisms and comparison with other branching systems. Mycologia.

[36] Cardoso C, Ruyter-Spira C, Bouwmeester HJ (2011). Strigolactones and root infestation by plant-parasitic *Striga*, *Orobanche* and *Phelipanche* spp. Plant Sci..

[37] Driss-Ecole D, Cottignies A, Jeune B, Corbineau F, Perbal G (1994). Increased mass production of *Veronica arvensis* grown on a slowly rotating clinostat. Environ. Exp. Bot..

[38] Dunser K, Kleine-Vehn J (2015). Differential growth regulation in plants—the acid growth balloon theory. Curr. Opin. Plant Biol..

[39] Kamal KY, Herranz R, van Loon JJWA, Medina FJ (2018). Simulated microgravity, Mars gravity, and 2g hypergravity affect cell cycle regulation, ribosome biogenesis, and epigenetics in *Arabidopsis* cell cultures. Sci. Rep..

[40] Al-Yahya’ei MN (2011). Unique arbuscular mycorrhizal fungal communities uncovered in date palm plantations and surrounding desert habitats of Southern Arabia. Mycorrhiza.

[41] Wuest SL (2015). Simulated microgravity: critical review on the use of random positioning machines for mammalian cell culture. Biomed. Res. Int..

[42] Kraft TF, van Loon JJ, Kiss JZ (2000). Plastid position in *Arabidopsis columella* cells is similar in microgravity and on a random-positioning machine. Planta.

[43] Hoson T, Kamisaka S, Masuda Y, Yamashita M, Buchen B (1997). Evaluation of the three-dimensional clinostat as a simulator of weightlessness. Planta.

[44] Hoson T (1999). Morphogenesis of rice and *Arabidopsis* seedlings in space. J. Plant Res..

[45] Jost AI, Hoson T, Iversen TH (2015). The utilization of plant facilities on the international space station-the composition, growth, and development of plant cell walls under microgravity conditions. Plants.

[46] Wuest, S. L., Stern, P., Casartelli, E. & Egli, M. Fluid dynamics appearing during simulated microgravity using random positioning machines. PLoS ONE 12 (2017) http://journals.plos.org/plosone/article?id=10.1371/journal.pone.0170826. 10.1371/journal.pone.0170826 PMC5279744 28135286

