# Supplementary material for: Simulated microgravity and the antagonistic influence of strigolactone on plant nutrient uptake in low nutrient conditions
Source: NPJ Microgravity. 2018 Oct 17;4:20. doi: 10.1038/s41526-018-0054-z (PMC6193021; doi:10.1038/s41526-018-0054-z)
Supplement: Supplementary file 2 — Supplemental Material [file 41526_2018_54_MOESM2_ESM.pdf]

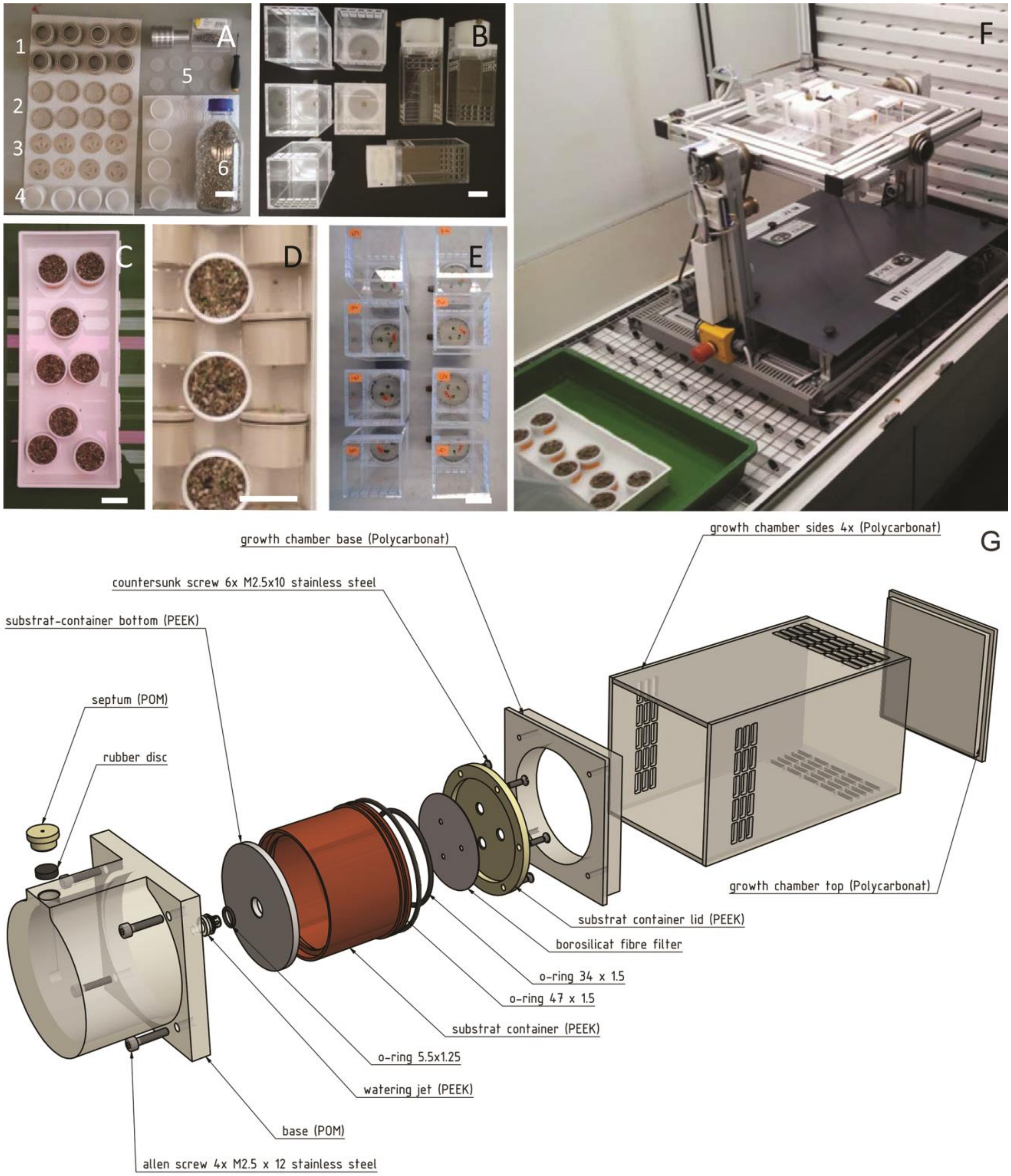

Supplementary Figure 1. Multigen-1 like chambers. A) Parts crafted to assemble eight Multigen-1-like (M1L) chambers: 1. pots 2. pot bottom closures 3. pot lids 4. mock-pots for *1-g* conditions 5. plastic mesh to contain the clay+AMF inoculum mixture in the pot under *s0-g* conditions 6. clay with AMF inoculum. B) The eight M1L chambers with mounted transparent Plexiglas,

growth chambers. C) Mock-pots placed in the growth chamber. D) Mock pots (top layer) and M1L pots (bottom layer) before being mounted on M1L chambers. E) *Petunia* seedlings growing in M1L chambers. F) Bottom left the *1-g* mock pots, on the right the RPM with M1L mounted chambers. G) Exploded scheme of one M1L chamber. Seedlings were grown in the substrate container filled with clay + AMF inoculum. Plants were watered through the septum with a syringe. The borosilicate fibre filter placed on the clay surface helped keeping the substrate in position while rotating on the RPM. The transparent growth chamber with openings on each side helped gas exchange and protected the seedlings from air movements generated by continuous rotation. Scale bars: A, C, D = 5 cm; B = 2 cm; E = 3 cm

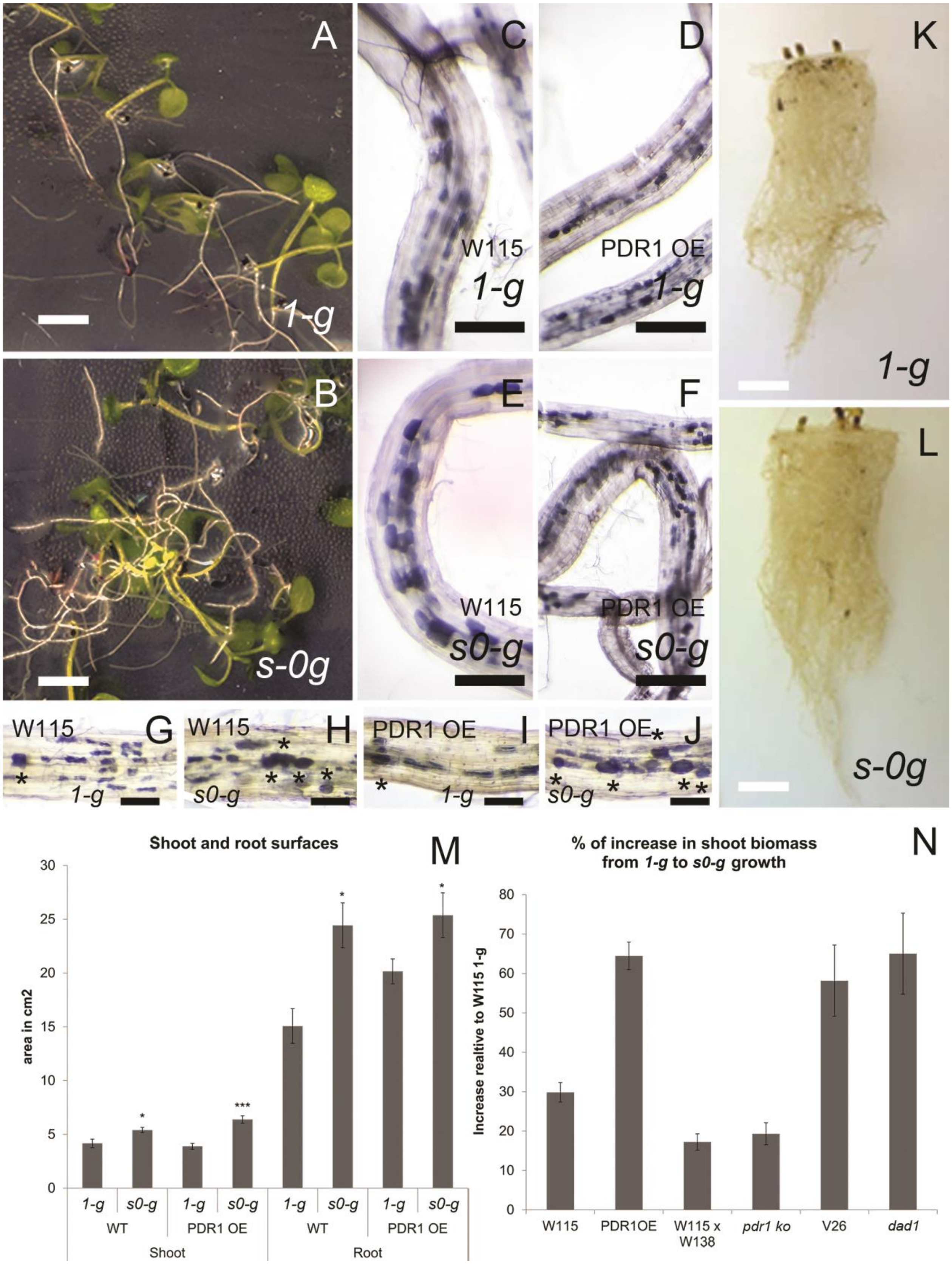
A
1-g
B
s-0g
C
W115
1-g
D
PDR1 OE
1-g
E
W115
s0-g
F
PDR1 OE
s0-g
G
W115
1-g
H
W115
s0-g
I
PDR1 OE
1-g
J
PDR1 OE
s0-g
K
1-g
L
s-0g
M
Shoot and root surfaces
area in cm2
0
5
10
15
20
25
30
*
***
*
*
1-g
s0-g
1-g
s0-g
1-g
s0-g
1-g
s0-g
WT
PDR1 OE
WT
PDR1 OE
Shoot
Root
N
% of increase in shoot biomass
from 1-g to s0-g growth
Increase realtive to W115 1-g
0
10
20
30
40
50
60
70
80
W115
PDR1OE
W115 x
W138
pdr1 ko
V26
dad1

Supplementary Figure 2. *Petunia* mycorrhization and biomass production in simulated microgravity. A, B) Test agar plates with *Petunia* seedlings: (A) at *1-g* and (B) *s0-g* conditions. C-J) Ink staining of AMF structures in colonized *Petunia* roots of W115 (WT) and PDR1 OE plants in *1-g* and *s0-g* conditions (stars = vesicles). K-L) Roots of wild-type W115 *Petunia* plants grown at *1-g* and *s0-g*. M) Shoot and root surfaces quantified via ImageJ. N) Percent of increase in shoot biomass per plant in *s0-g* compared to *1-g* conditions. Scale bars: A, B = 1 cm; C-F = 710 μm; G-J = 280 μm; K, L= 1 cm. Error bars are SE.

Supplementary Video 1. Simulated microgravity operated with a Random Positioning Machine to test mycorrhizal development and plant biomass production

Supplementary Table 1 Statistical analyses

Figure		Description	n	T-Test p	notes
Fig. 1	G	W115 1-g vs s0-g	5	4.60E-02	500 intersections per replicate
		PDR1 OE 1-g vs s0-g	5	3.28E-02	500 intersections per replicate
	H	W115xW138 1-g vs s0-g	5	3.70E-03	500 intersections per replicate
		pdr1 ko 1-g vs s0-g	5	1.90E-03	500 intersections per replicate
Fig. 2	A	WT 1-g vs s0-g	3	7.90E-03	3 plants per replicate
		PDR1 OE 1-g vs s0-g	3	2.29E-02	3 plants per replicate
		pdr1 ko 1-g vs s0-g	3	8.10E-03	3 plants per replicate
	O	WT 1-g vs s0-g	2	1.01E-02	9 plants per replicate
		PDR1 OE 1-g vs s0-g	2	8.50E-03	9 plants per replicate
Fig. 4	E	full stem 1-g vs s0-g	5	3.30E-03	
		pith 1-g s0-g	5	4.60E-03	
	F	1-g vs s0g	5	4.24E-05	10 cells per slice
	I	leaf5 1-g vs s0-g	12	7.02E-03	
		leaf 6 1-g vs s0-g	12	4.25E-02	
		leaf7 1-g vs s-0g	12	4.06E-02	
		leaf8 1-g vs s0-g	12	3.41E-02	
Fig. S2	M	WT 1-g vs s0-g	9	2.61E-02	
		PDR1 OE 1-g vs s0-g	9	3.38E-05	
		WT 1-g vs s0-g	9	7.25E-03	
		PDR1 OE 1-g vs s0-g	9	4.10E-02	

n equals to biological replicates. All statistical tests are two-sided.
